# Vector-borne diseases and climate change: a European perspective

**DOI:** 10.1093/femsle/fnx244

**Published:** 2017-11-15

**Authors:** Jan C Semenza, Jonathan E Suk

**Affiliations:** European Centre for Disease Prevention and Control, Tomtebodavägen 11A, Stockholm, S-171 83, Sweden

**Keywords:** climate change, vector-borne diseases, Zika, dengue, chikungunya, Leishmaniasis

## Abstract

Climate change has already impacted the transmission of a wide range of vector-borne diseases in Europe, and it will continue to do so in the coming decades. Climate change has been implicated in the observed shift of ticks to elevated altitudes and latitudes, notably including the *Ixodes ricinus* tick species that is a vector for Lyme borreliosis and tick-borne encephalitis. Climate change is also thought to have been a factor in the expansion of other important disease vectors in Europe: *Aedes albopictus* (the Asian tiger mosquito), which transmits diseases such as Zika, dengue and chikungunya, and *Phlebotomus* sandfly species, which transmits diseases including Leishmaniasis. In addition, highly elevated temperatures in the summer of 2010 have been associated with an epidemic of West Nile Fever in Southeast Europe and subsequent outbreaks have been linked to summer temperature anomalies. Future climate-sensitive health impacts are challenging to project quantitatively, in part due to the intricate interplay between non-climatic and climatic drivers, weather-sensitive pathogens and climate-change adaptation. Moreover, globalisation and international air travel contribute to pathogen and vector dispersion internationally. Nevertheless, monitoring forecasts of meteorological conditions can help detect epidemic precursors of vector-borne disease outbreaks and serve as early warning systems for risk reduction.

## Teaser

The impact of climate change on vector-borne diseases has implications for public health and calls for monitoring climatic precursors of outbreaks as well as integrated surveillance of cases and vectors.

## BACKGROUND

The transmission of vector-borne diseases requires an introduced and/or established vector population, a pathogen and suitable environmental and climatic conditions across the full cycle of vector-borne disease transmission in humans (Randolph and Rogers 2010). The latter affect everything from vector survival and abundance, pathogen growth and survival in host organisms and in vector organisms, vector activity and biting rates and human exposures to disease vectors.

Although climate change is the focus of this paper, it is important to stress that the introduction of exotic diseases and disease vectors in Europe is primarily facilitated by globalisation (Semenza *et al*. 2016a). Trade and travel in particular can increase the importation risks. Global air travel and seaborne trade appear to facilitate the expansion of invasive mosquito species (Tatem, Hay and Rogers 2006). Human migration, while leading occasionally to large-scale population movements, has not been shown to lead to an increased risk of disease spread with ‘host’ populations in Europe (Semenza *et al*. 2016b). One consequence of globalisation is that in a highly interconnected world, seemingly unrelated factors from disparate locations may combine to contribute to the generation of novel infectious disease risks (Suk *et al*. 2014a). A notable example of this in continental Europe was the detected outbreaks of Chikungunya, identified in Italy in 2007 and 2017 (Rezza *et al*. 2007; Venturi *et al*. 2017), which were, in part, due to global trade in tires that enabled the introduction of *Aedes albopictus* mosquitos into Europe, a permissive climate for the expansion of these mosquitos in Europe, and global air traffic that subsequently led to the introduction of the virus in a region where the mosquito was present and active. Myriad additional ecological and socio-economic drivers are important contributors to the establishment and ultimate transmission of vector-borne diseases, including land use and farming practices, fauna, public health capacities and human exposure to vectors (Jones *et al*. 2008; Suk and Semenza 2011; McMichael 2013; Semenza *et al*. 2016a, 2016c).

Climate change, meanwhile, most predominantly affects seasonal range expansions and contractions of vector-borne diseases in Europe (Semenza and Menne 2009), although the lack of historic surveillance data constrains direct attributions to climate change (Randolph and Rogers 2010; Altizer *et al*. 2013; Rodó *et al*. 2013; Ostfeld and Brunner 2015; Parham *et al*. 2015; Ebi *et al*. 2017). Climate influences the life cycle of vectors, as well as the reproduction rate of parasites and viral particles inside vectors and human hosts (Semenza and Menne 2009), which means that upsurges in temperature can reduce the incubation period of these pathogens and the life cycle of vectors, thus boosting transmission risk through elevated vector populations, though within a certain temperature envelope. Long-term changes in the seasons can also affect vector and host animals, human activity and land use, which consequently could further affect the spatial-temporal distribution and prevalence of vector-borne diseases in Europe (Lindgren *et al*. 2012).

In this paper, we summarise the state of knowledge relating to the observed and projected impacts of climate change on vector-borne disease transmission in Europe, focusing on tick-borne, mosquito-borne and sandfly-borne diseases, before identifying paths forward for research and public health action.

## TICK-BORNE DISEASES

### Past trends

In Europe, *Ixodes ricinus* is the primary vector for both Lyme borreliosis and tick-borne encephalitis (TBE), the most important tick-borne diseases. With an estimated 65 000 cases a year, Lyme borreliosis is responsible for the largest disease burden of any vector-borne disease in the European Union (EU).^1^ In 2014, 2057 cases of TBE were notified in the EU. There has been a nearly 400% increase of reported cases in European endemic areas over the past 30 years, although this number is also due to enhanced surveillance and diagnosis (Medlock *et al*. 2013; ECDC 2014).

**Figure 1.**
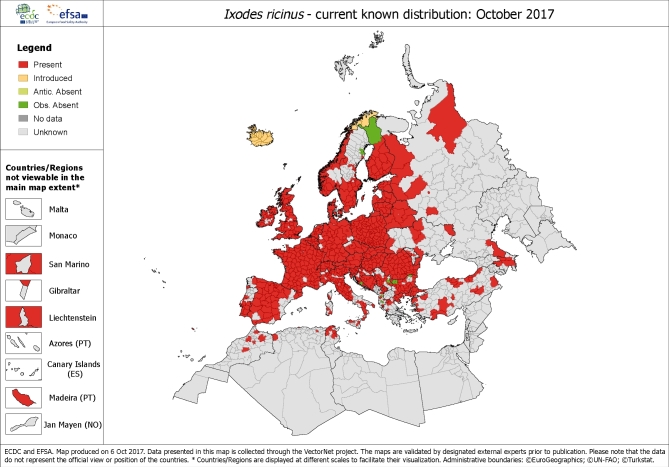
Current distribution of *I. ricinus* ticks in Europe. The resolution assst ‘regional’ administrative level (NUTS3) is based on published historical data provided by technical countries experts as part of the ECDC VectorNet project. Distribution is most likely to be under-reported as notification is not mandatory. Source: Copyright © European Centre for Disease Prevention and Control and European Food Safety Authority.

A necessary but not sufficient determinant of disease incidence is the presence and abundance of ticks; however, the distribution of ticks and the observed incidence of TBE differ considerably (Süss *et al*. 2006). Ticks are susceptible to climatic determinants, specifically humidity and temperature. *Ixodes ricinus* is present throughout a large part of continental Europe (Fig. 1) and there has been a documented expansion to higher latitudes and altitudes, with reports of movement northerly in Sweden (Jaenson *et al*. 2012) and to higher elevations in Austria and the Czech Republic (Daniel *et al*. 2003; Heinz *et al*. 2015). Range expansions have also been described in Norway and Germany (Semenza and Menne 2009).

High incidence of tick-borne disease has been reported to be linked with moderate winters and humid, warm summers in Sweden, Slovakia and Hungary, although incidence may also be affected by the influence of climate on recreational activities (Ostfeld and Brunner 2015). Lyme disease risk has been linked to warm winters, elevated summer temperatures, low seasonal temperature variation and high vegetation indices (Estrada-Pena *et al*. 2011). For TBE, the relative importance of climate change *vis a vis* other factors varies by location and is a function of immunisation coverage, tourism activity, human exposure, rodent host population density and socio-economic conditions (Randolph 2008).

There are incomplete data regarding climate change and other tick-borne diseases. Some studies have indicated that the Mediterranean basin is becoming more hospitable for the spread of Crimean–Congo haemorrhagic fever (Maltezou and Papa 2010), but socio-economic development, agricultural practices and land use changes may be more significant contributors (Estrada-Peña *et al*. 2012). There has been a range expansion of Rickettsia in recent years, but the underlying reasons are not clear (Gouriet, Rolain and Raoult 2006) and few investigations have examined the association between climate change and this disease.

### Future projections

The altitudinal and latitudinal limits of *I. ricinus* seem to be constrained by cold temperature (Ostfeld and Brunner 2015), and a range expansion of ticks to higher altitudes and latitudes is projected. Under climate-change scenarios, warmer winter temperatures, longer growing seasons and earlier summers with elevated temperatures are projected to occur, which could also result in a shift in the distribution of deer host populations (Jaenson and Lindgren 2011).

By 2040–2060, a 3.8% overall habitat enlargement for *I. ricinus* is anticipated in Europe under one climate projection, with extension into higher altitudes and latitudes in some areas, especially in Scandinavian and Baltic countries. In contrast, a contraction is foreseen in the Alps, Pyrenees, north-western Poland and the interior of Italy (Boeckmann and Joyner 2014). This observation is consistent with other projections of climate change that predict an expansion of the *I. ricinus* range (Estrada-Peña, Ayllón and de la Fuente 2012; Porretta *et al*. 2013) although it is important to note that in these models many uncertainties exist in inferring the projected habitat ranges to projected tick-borne disease incidence.

Nevertheless, TBE incidence is generally projected to move to higher altitudes and latitudes in line with the distribution of *I. ricinus*, resulting in an increased risk in some parts of northern and central Europe (notwithstanding the potential implementation of targeted vaccination programmes and enhanced TBE surveillance). At the same time, the risk of TBE is largely anticipated to diminish in the south of Europe. Likewise, milder winters may enable the extension of Lyme borreliosis to higher altitudes and latitudes, predominantly in the north of Europe, but the risk is expected to diminish in areas of Europe that are projected to suffer extended dry spells (Semenza and Menne 2009).

## MOSQUITO-BORNE DISEASES

### Past trends

Locally transmitted epidemics of malaria, dengue and chikungunya have occurred in continental Europe over the past decade (ECDC 2014).

**Figure 2.**
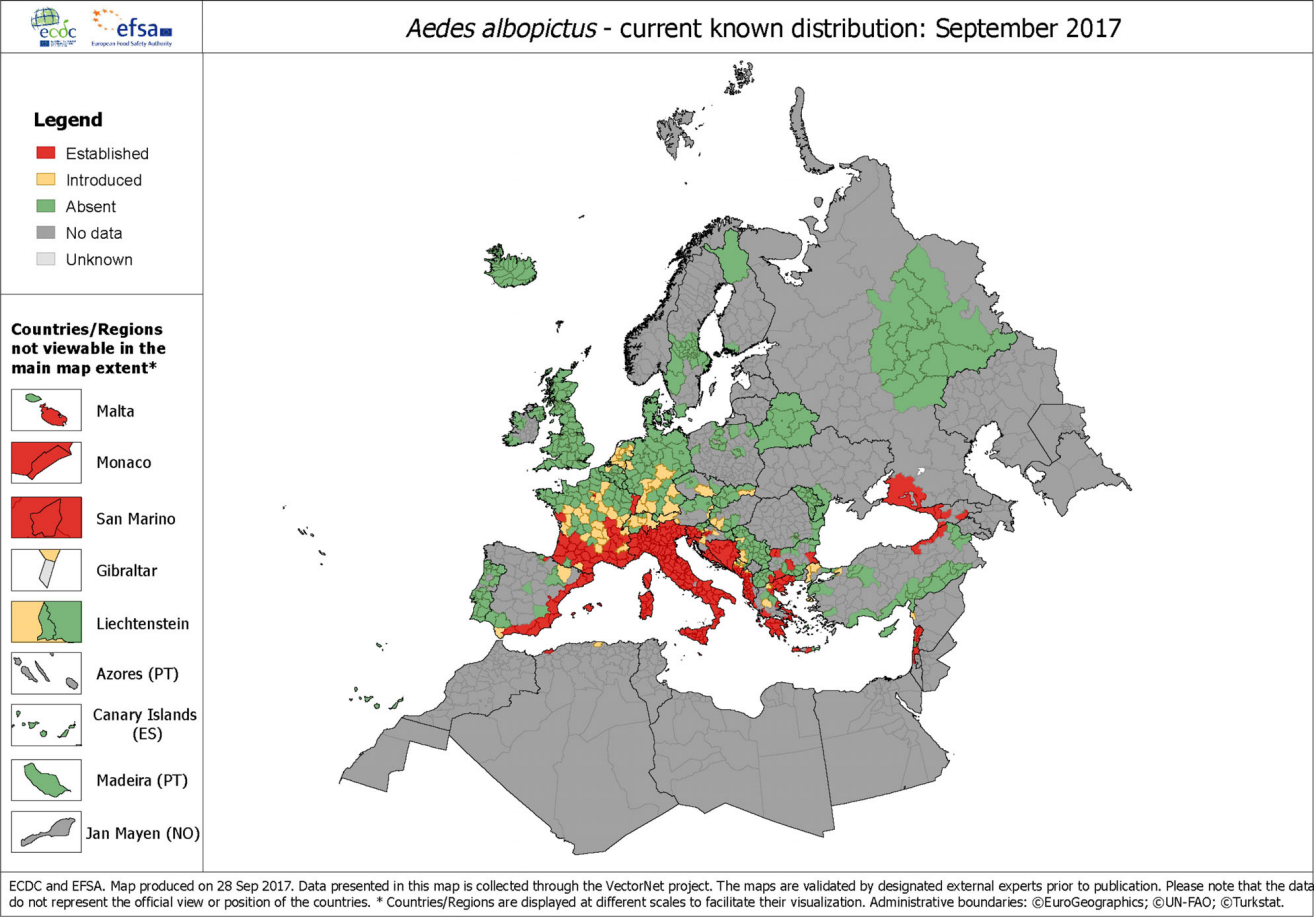
Current distribution of *A. albopictus* in Europe. Distribution *A. albopictus* at ‘regional’ administrative level (NUTS3) in Europe, based on confirmed, unpublished and published data made available by national entomologists participating in VectorNet. Please note that the vector presence is likely to be under-reported as reporting is not mandatory. Key: RED: *A. albopictus* populations are established and there is evidence of reproducing and overwintering mosquitoes in at least one location in the administrative region. YELLOW: *A. albopictus* mosquitoes have been introduced but are not reported to be established over the past 5 years in the administrative region. DARK GREEN: *A. albopictus* mosquitoes have not been reported to be introduced or established in the past 5 years despite field surveys and other entomological investigations. MEDIUM GREY: Lack of data over the past 5 years that are available to national entomologists. LIGHT GREY: No field entomological filed investigations over the past 5 years. Source: http://ecdc.europa.eu/en/activities/diseaseprogrammes/emerging_and_vector_borne_diseases/Pages/VBORNET.aspx online. Copyright © European Centre for Disease Prevention and Control, 2017.

*Aedes albopictus, a*lso known as the Asian tiger mosquito, can transmit dengue, chikungunya and Zika. The world's most invasive mosquito, it became established in Italy in 1990 and subsequently spread to several other EU and neighbouring countries with a particularly strong presence in the Mediterranean basin (Benedict *et al*. 2007; Fig. 2).

**Figure 3.**
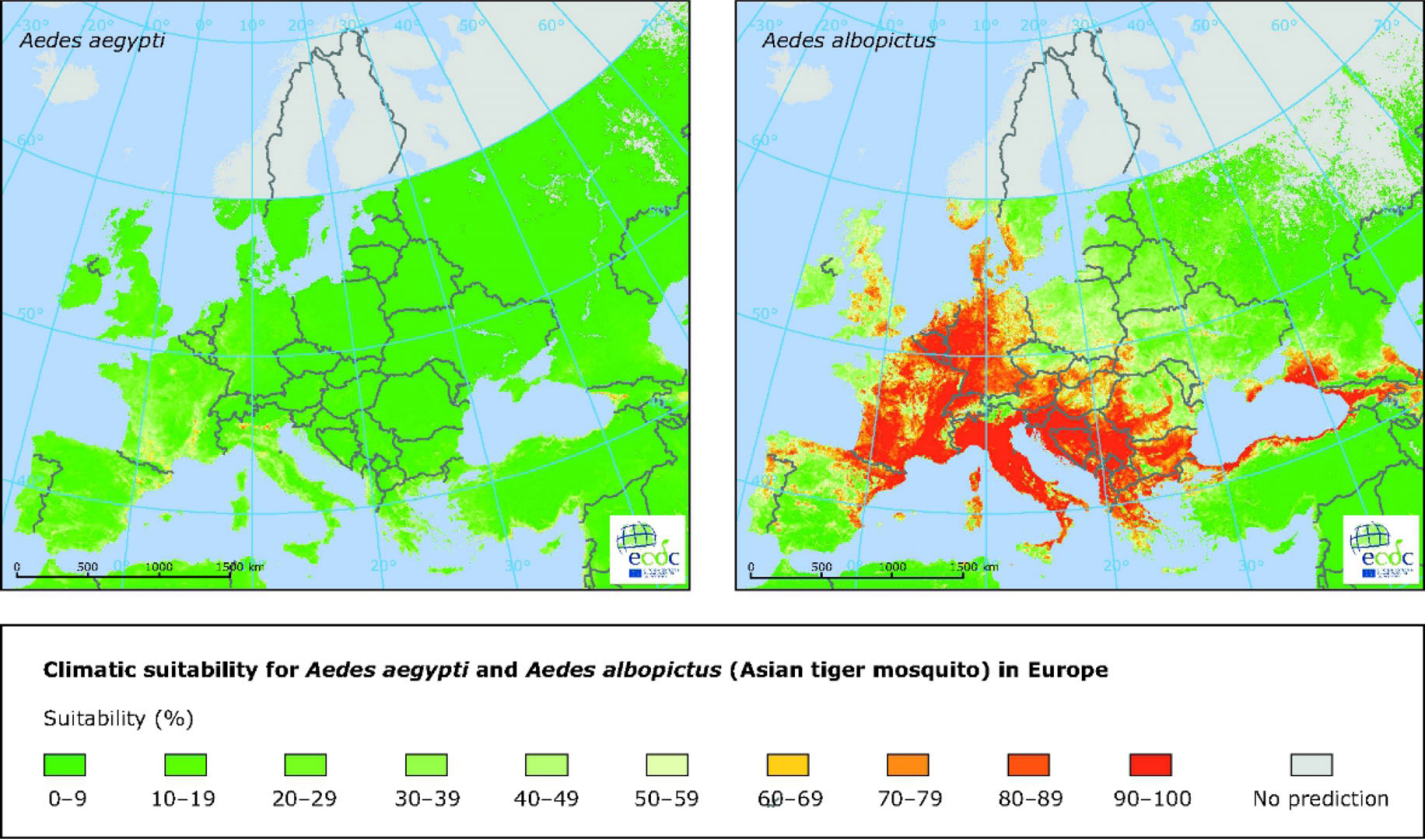
Climatic suitability for the mosquitoes *Aedes aegypti* and *A. albopictus* in Europe. Note: Yellow to red designates conditions that are increasingly suitable for the vector whereas darker to lighter green designates conditions not suitable for the vector. Grey indicates that no prediction is possible. Source: Copyright © European Centre for Disease Prevention and Control, 2012.

Although trade and travel played a role in the introduction and subsequent dispersion of the vector, climate change is also believed to have been a factor, despite that its relative contribution is uncertain (Caminade *et al*. 2012). Warm seasonal and annual temperatures and ample rainfall in Europe offer conducive climatic conditions for *A. albopictus* (Roiz *et al*. 2011). The introduction and geographical expansion of the vector has coincided with favourable climatic conditions in France, the Balkans, the eastern coasts of Spain and the Adriatic Sea, and the Benelux countries and western Germany (Caminade *et al*. 2012). However, some other parts of Europe that have not yet observed the presence and establishment of the vector are climatically suitable for *A. albopictus* (Fig. 3; Rogers, Suk and Semenza 2014; Proestos *et al*. 2015).

A number of epidemics caused by the *A. albopictus* mosquito have been observed in Europe, including several autochthonous chikungunya outbreaks in France and Italy in 2007, 2010, 2014, 2015 and 2017, and autochthonous dengue outbreaks in France and Croatia in 2010 (Rezza *et al*. 2007; La Ruche *et al*. 2010; Gjenero-Margan *et al*. 2011; Grandadam 2011; Delisle *et al*. 2015; Venturi *et al*. 2017). Heavy precipitation may have contributed to the autochthonous chikungunya transmission in France in 2014 by causing an explosive increase in vector abundance (Roiz *et al*. 2011; ECDC 2015).

Increased geospatial dispersion of *A. albopictus* in Europe also increases the risk of onward transmission of dengue, chikungunya and Zika via returning travellers (Semenza *et al*. 2014). For example, this risk is likely to have increased for chikungunya subsequent to the extended epidemic in late 2013 that started in the Caribbean and engulfed many countries of the American region (Van Bortel *et al*. 2014).

Although certain regions of Europe are climatically hospitable to *Aedes aegypti*, the primary vector for dengue, the risk is currently rather low, as this primary vector is not established in Europe and vector control measures are established (ECDC 2012). However, *Aedes aegypti* has been implicated for occasional dengue epidemics in Europe, including the epidemic that affected Madeira, Portugal in 2013 (ECDC 2013).

Malaria, meanwhile, was eradicated from Europe in 1975 but its vectors (*Anopheles* mosquitoes) are still widely established throughout; in fact, each year a few isolated cases of autochthonous transmission occurs (Florescu *et al*. 2011). The risk of re-establishment is a function of a number of determinants, including climatic, ecological, land use and socio-economic factors. An outbreak of malaria caused by *P. vivax* erupted in Greece in 2009 and continued in the subsequent years due to elevated temperature, agricultural and land-use practices (Sudre *et al*. 2013). Nevertheless, malaria risk remains very low in Europe due to economic and social development and access to health care (Gething *et al*. 2010).

**Figure 4.**
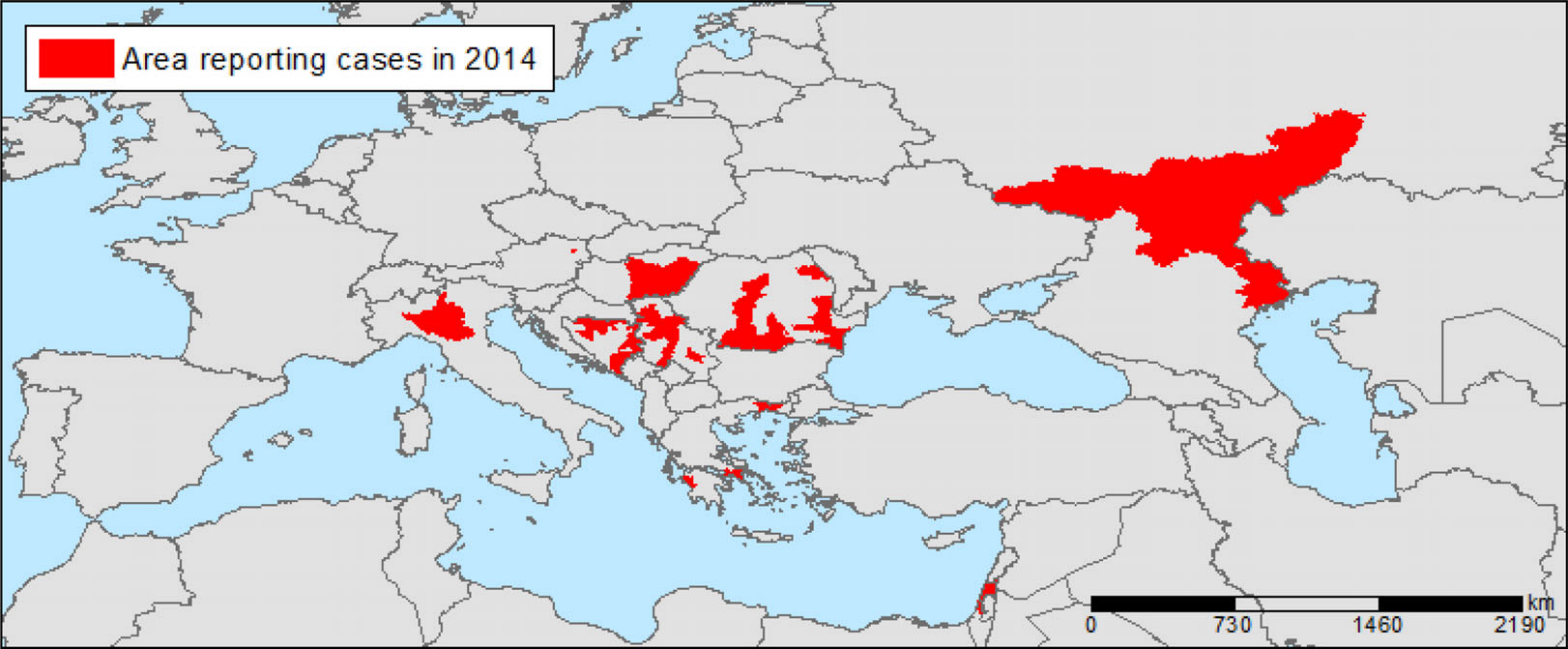
Reported West Nile Virus infections, 2014. Confirmed and probable cases of West Nile Virus infections by district, as of 20/11/2014. Source: Semenza *et al*. 2016

West Nile Virus (WNV) infections are transmitted through *Culex* sp. mosquito. Infections can be rather serious, mainly among the elderly, although over 60% of infections remain asymptomatic. Infections can also be acquired through contaminated substances of human origin (donated blood, transplants, etc.) and, while infrequent, such instances have been described (Petersen, Brault and Nasci 2013). Starting in 2010, there have been yearly epidemics of WNV in eastern and southern Europe, providing evidence of ongoing transmission (Fig. 4; Paz and Semenza 2013; Semenza 2015). Elevated temperature anomalies from the monthly average were a significant determinant of the 2010 WNV outbreak (Paz *et al*. 2013). In subsequent years, other environmental factors, such as the state of vegetation, water bodies and bird migratory routes were also identified as important drivers (Tran *et al*. 2014; Marcantonio *et al*. 2015). July temperature anomalies were used as a predictor of WNF risk later on in the season (Semenza 2015).

### Future projections

In the regions where climatic models project wetter and warmer conditions, the climatic suitability is projected to intensity for *A. albopictus*, including the south and east of the United Kingdom (Medlock and Leach 2015), the Balkans and central Europe. In contrast, suitability is largely expected to diminish in regions where the climate is projected to become drier, such as in some areas of Portugal and Spain (Caminade *et al*. 2012). This is in agreement with an analysis that found a reduction in the habitat suitability in the Mediterranean region and in southern Europe, and an expansion of the habitat suitability in eastern and northern Europe (Proestos *et al*. 2015).

**Figure 5.**
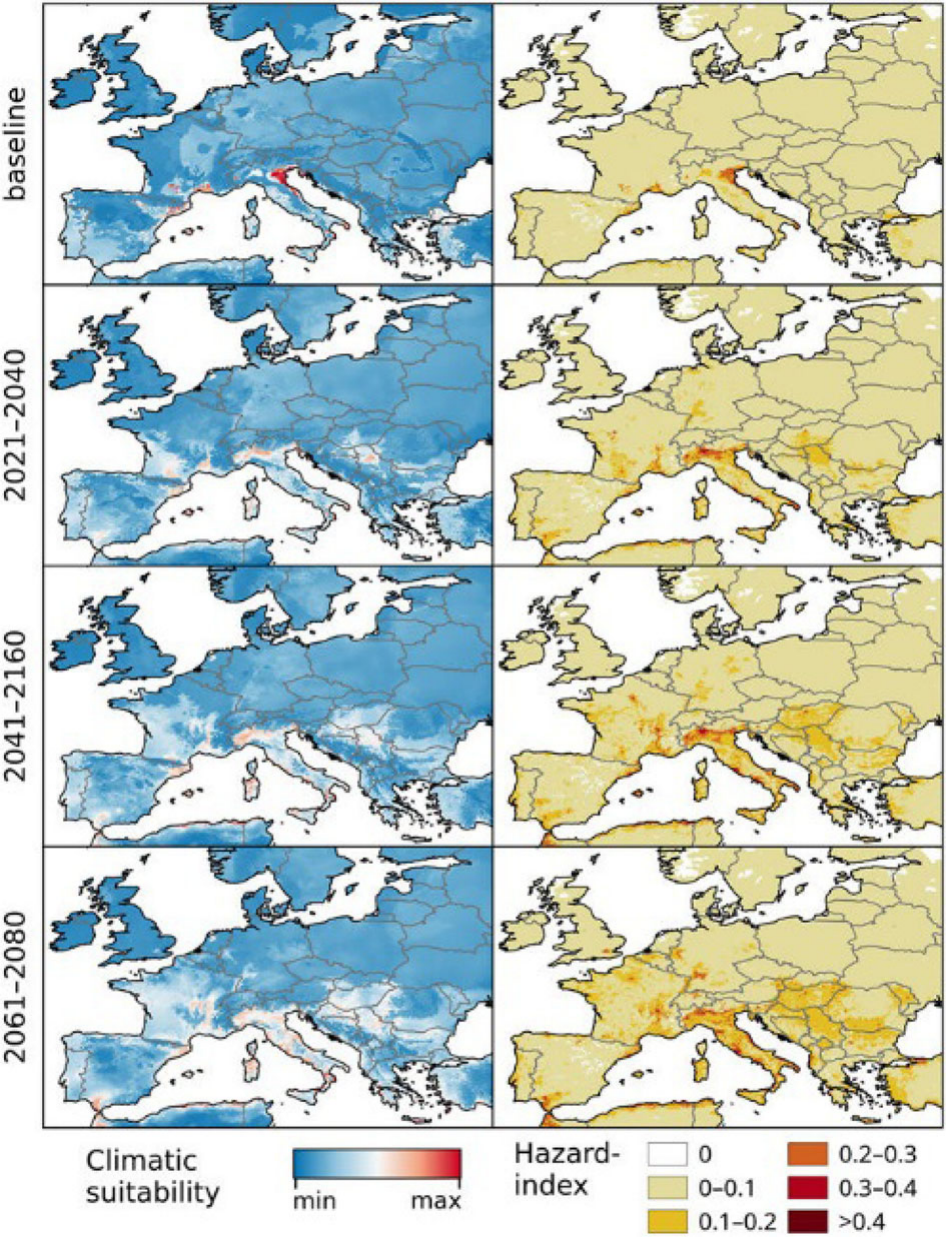
Chikungunya under the baseline and Representative Concentration Pathway (RCP) 8.5 climate-change scenarios in Europe. The RCP 8.5 climate-change scenario anticipates high energy demands and greenhouse gas emissions (see Riahi *et al*. 2011). Left: climatic suitability, right: hazard index. Climate-change scenarios represent the mean model output obtained through the five general circulation models. Climatic suitability output is scaled to the over-all global minimum (0) and maximum (0.623) values observed in any model. Maps were generated using the ‘raster’ package in R 3.3.2 (https://www.r-project.org/) and QGIS 2.8.1 (https://www.qgis.org/). Source: (Tjaden *et al*. 2017, Fig. 5).

Importation risk due to air travel and local transmission is increasing in European areas where the seasonal abundance of *A. albopictus* matches the seasonality of chikungunya epidemics in countries outside of Europe, regardless if perpetuated by *Aedes aegypti* or *A. albopictus* (Charrel, de Lamballerie and Raoult 2008). Thus, with the rapid expansion of chikungunya worldwide, the risk for Europe may have increased. For example, models have generally projected that a moderate climatic suitability for chikungunya transmission is anticipated, notably across France, Spain, Germany and Italy (Nsoesie *et al*. 2016; Tjaden *et al*. 2017). There are some regional variations, however, with increased suitability projected for large areas by the Rhine and Rhone rivers, while some areas by the Adriatic coast in Italy are projected to experience a decline in suitability due to the increased probability of summer droughts (Tjaden *et al*. 2017). The latter finding also corresponds with previous work that has suggested that the risk of Chikungunya will diminish in the Mediterranean region despite remaining climatically suitable for chikungunya transmission (Fig. 5; Fischer *et al*. 2013).

The risk for dengue in Europe could increase due to climate-related proliferation in the density or seasonal activity of *A. albopictus*. The risk could also potentially increase if variations in temperatures enable the re-establishment of *Aedes aegypti*, but further modelling studies for continental Europe are needed to determine whether climate change would decrease or increase the climatic suitability for *Aedes aegypti*.

Malaria models for continental Europe suggest elevated suitability for malaria transmission due to climate change, even though projected transmission areas of malaria are very sensitive to the input variables (Caminade *et al*. 2014). Vector abatement strategies, land use and socio-economic development should be adequate to contain the malaria risk at the edges of its range, in spite of the probability of air passenger introductions (Semenza *et al*. 2014).

**Figure 6.**
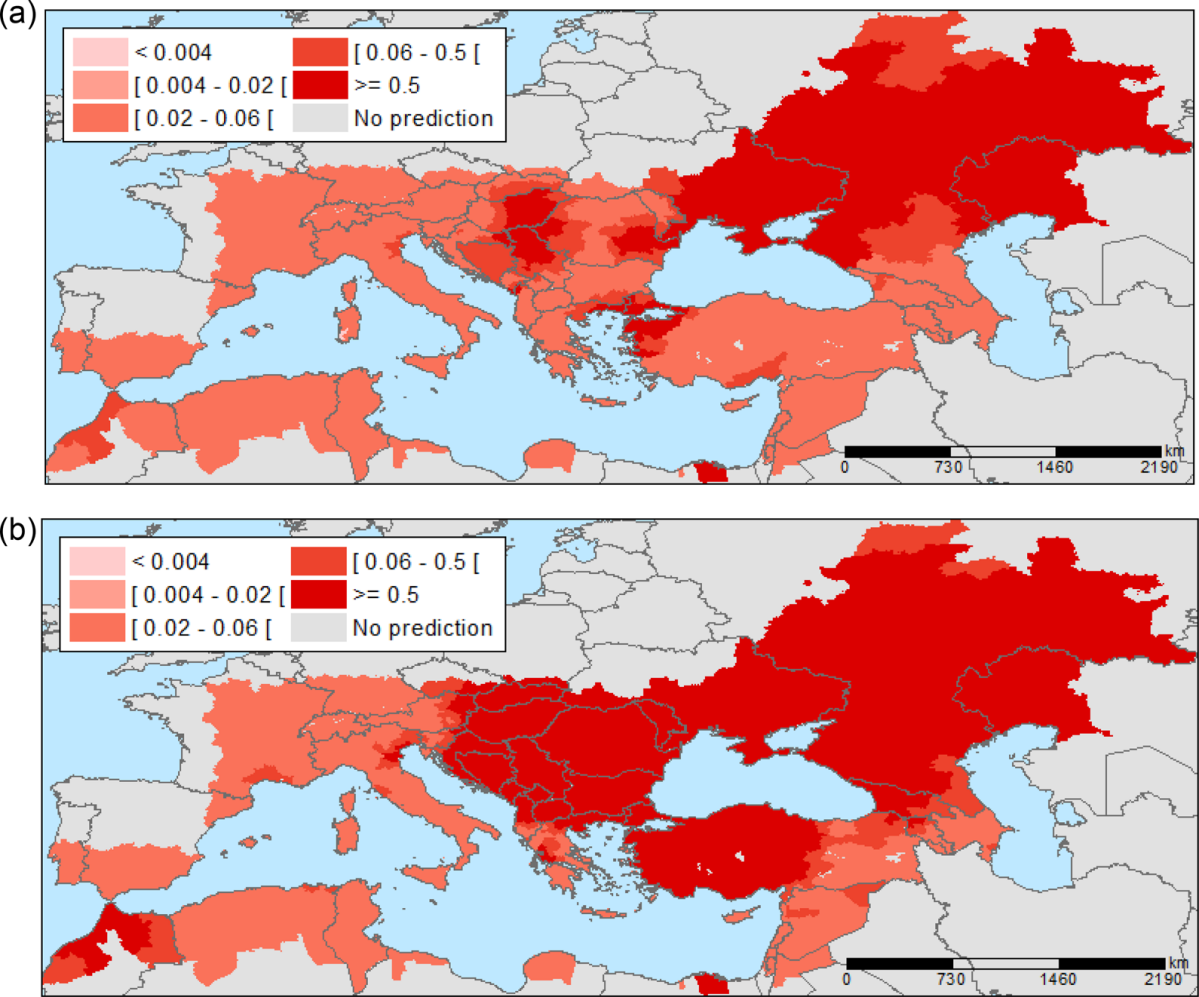
West Nile Virus infections: projected future distribution in Europe. Projected probability of districts with West Nile Virus infections for 2025 (**a**) and 2050 (**b**), based on July temperatures for A1B scenario projections (a scenario of rapid economic growth, global population peaking by mid-21st Century, and rapid introduction of new and more efficient energy technologies). Source: (Semenza *et al*. 2016).

Temperature anomalies due to climate change might influence WNV transmission in Europe by altering the geographic range of vectors, the aerial migration routes of avian WNV hosts and the pathogen life cycle. With July temperature projections for Europe under climate-change models for a medium emissions scenario (the A1B scenario of the Special Report on Emissions Scenarios describes a world of rapid economic growth, a global population peaking by mid-21st Century and the rapid introduction of new and more efficient energy technologies (IPCC 2000)), the WNV risk was projected to 2025 and 2050, keeping other variables constant (e.g. bird migratory routes, water index and state of vegetation; Semenza *et al*. 2016). The projections indicate a continuous extension of regions with an increased risk of WNV infections, mainly at the fringes of the regions of transmission (Fig. 6). Projections for 2025 show an elevated risk in north-east Greece, east Croatia and north-west Turkey; projections for 2050 show a further expansion of high-risk areas.

## SANDFLY-BORNE DISEASES

### Past trends

In Europe, leishmaniasis is the most prevalent disease transmitted by *phlebotomine* sandflies, which is caused by two parasites: *Leishmania infantum*, responsible for visceral leishmaniasis, and *Leishmania tropica*, responsible for cutaneous leishmaniasis. In the Mediterranean area, *L. infantum* is endemic, while *L. tropica* arises periodically in Greece and neighbouring countries. The transmission of these two parasites is highly influenced by temperature.

At present, *phlebotomine* sandflies have broader range than the parasites. In Europe, support for the contribution of climate change on the distribution of sandflies is limited (Ready 2010), although in Italy climate change has been suspected as a potential factor for the documented northward shift of sandfly vectors (Maroli *et al*. 2008). For central Europe, the current risk is projected to be low due to temperature restrictions on pathogen reproduction (Fischer, Thomas and Beierkuhnlein 2010).

### Future projections

Temperature and relative humidity affect the survival and reproduction rate of sandlfies (Negev *et al*. 2015) and parasite development, and thus climate change could shift the range of leishmaniasis in the future. In some regions of southern Europe, the risk of disease transmission may decrease where climate conditions become too hot and too dry for vector survival. One projection suggests that the climate in central Europe will increasingly become hospitable for *Phelobotomus spp*. sandflies (Fischer *et al*. 2011). Another modelling study predicted that, by the end of the 2060s, the southern United Kingdom, France, Germany, and western Poland could be populated by sandflies, predominantly *P. ariasi and P. pernicious*, whilst the Balkan Peninsula, Mediterranean Basin and Carpathian Basin could be climatically hospitable for many *Phlebotomus* species (Trájer *et al*. 2013).

Expanded climatic suitability for sandflies could extend leishmaniasis risk, although expansion may be contained by the somewhat restricted movement ability of sandflies.

## FUTURE DIRECTIONS AND ACTIVITIES

One recent study identified the climate sensitivity of significant human and domestic animal pathogens in Europe, noting that 63% were climate sensitive (McIntyre *et al*. 2017). Despite these strong associations, there remain important gaps in studies that attempt to project the impact of climate change on future vector-borne disease transmission. As many other reviews have noted, main gaps include better parameterising the relationships between climatic variables and the key biological processes related to vector-borne disease transmission (e.g. Altizer *et al*. 2013; Parham *et al*. 2015). Downscaled climate-impact models focused on the European continent could, meanwhile, provide higher resolution on areas with projected changes in risk profiles. In addition, more detailed knowledge of the relationships between climatic drivers and vector-borne disease transmission could facilitate the development and deployment of early warning systems that integrate climatic and epidemiologic data (Lindgren *et al*. 2012; Semenza and Zeller 2014).

A cornerstone for projection modelling studies is long-term historical data. Thus, another priority for the field is to assemble as detailed as possible data on the presence and absence of important disease vectors (and, ideally, disease reservoirs). One such initiative is VectorNet, a joint initiative of the European Food Safety Authority and European Centre for Disease Prevention and Control, which collects data on ticks, mosquitoes, sandflies and biting midges in Europe.

Just as VectorNet collections information relevant for both animal and human health, progress in the field more generally requires greater collaboration between ecologists, virologists, microbiologists, entomologists and stakeholders from the food, animal and human health sectors. In addition, a fully integrated perspective on climate change and vector-borne disease must also develop a more holistic understanding of risk by accounting for vulnerabilities (Suk *et al*. 2014b). As observed elsewhere, socioeconomic contexts can have a profound impact on how and whether the risks from climate change manifest themselves (Parham *et al*. 2015; Suk 2016): ecological niche models, for example, only present half of the story. A truly integrated understanding would also account for human vulnerabilities. For example, during the 2011 outbreak of Malaria in Greece, migrant agricultural workers played a role in introducing the disease and were also among the most vulnerable to infection, due to limited access to healthcare, poor living conditions close to mosquito breeding sites and suboptimal malaria awareness (Evlampidou *et al*. 2015; Sudre *et al*. 2013). Vector-borne disease transmission occurs due to both climatic and socioeconomic factors, and it is important to avoid climate determinism in studies assessing the future impact of climate change.

Finally, moving forward, it will be essential to ensure that there is increased health sector engagement about the potential risks from climate change. This includes developing knowledge among public health practitioners on how to conduct, interpret and assess climate-change attribution and impact studies. With increased awareness, more momentum could be gathered to develop long-term and cross-sectoral preparedness strategies that systematically account for the changing dynamics of vector-borne disease transmission that can be expected in the coming decades.

## CONCLUSION

Climate change is projected to lead to a further shift of specific tick species to higher latitudes and altitudes and to continue to play a role in the expansion of geographical distribution of the *A. albopictus* mosquito and of sandfly species in Europe. Integrated surveillance of human cases and invasive and endemic mosquito species will be a cornerstone for effective prevention and control of vector-borne diseases (Semenza and Zeller 2014). Moreover, monitoring environmental and climatic precursors of vector-borne diseases can help to anticipate a potential upsurge of cases. For example, July temperatures anomalies can be considered a precursor for WNV transmission later on in the season. Forecasts and predictions can be developed by linking the monitoring of these environmental/climatic precursors to dedicated disease surveillance systems with integrated vector surveillance (Semenza 2015). By intercepting the emergence and spread of vector-borne diseases under climate-change scenarios the human and financial costs of a potential epidemic can be contained.

***Conflict of interest*.** None declared.

## Footnotes

1 There is no standardised case definition or diagnosis for Lyme disease in Europe, so this number represents only a best estimate.
